# Supplementary material for: A novel ATP dependent dimethylsulfoniopropionate lyase in bacteria that releases dimethyl sulfide and acryloyl-CoA
Source: eLife. 2021 May 10;10:e64045. doi: 10.7554/eLife.64045 (PMC8163506; doi:10.7554/eLife.64045)
Supplement: Supplementary file 1. — (a) Information of the Antarctic samples used in this study. (b) Homology alignment of proteins in Psychrobacter sp. D2 with known DMSP lyases. (c) Kinetic parameters of DMSP lyases and DMSP demethylase DmdA. (d) Crystallographic data collection and refinement parameters of DddX. (e) Homology alignment of proteins in Psychrobacter sp. D2 with known enzymes involved in acrylate catabolism. (f) Strains and plasmids used in this study. (g) Composition of the basal medium (lacking the carbon source). (h) Primers used in this study. [file elife-64045-supp1.docx]

**Supplementary file 1a.** Information of the Antarctic samples used in this study.

| **Latitude (S)** | **Longitude (W)** | **Sample description** | **Sampling date** | **Number of isolated strains** |
| --- | --- | --- | --- | --- |
| 62°13′50″ | 58°58′15″ | Penguin feces, red-pigmented, agglomerated. | January 9, 2017 | 23 |
| 62°11′28″ | 58°55′0.5″ | Rotten red algae | January 10, 2017 | 11 |
| 62°11′52″ | 58°59′39″ | Intertidal sediments, soil, dark brown, muddy. | January 10, 2017 | 34 |
| 62°14′50″ | 58°58′37″ | Membrane sample filtered from 1.5 L surface seawaters | January 31, 2017 | 64 |
| 62°13′12″ | 58°51′42″ | Membrane sample filtered from 2.0 L surface seawaters | January 31, 2017 | 43 |

**Supplementary file 1b.** Homology alignment of proteins in *Psychrobacter* sp. D2 with known DMSP lyases.

| **Enzymes involved in DMSP catabolism** | **Proteins of the highest similarity in *Psychrobacter*.** **sp.** **D2** | **Similarity** |
| --- | --- | --- |
| DddD (*Marinomonas* sp. MWYL1) | orf01588 ([CoA transferase](https://blast.ncbi.nlm.nih.gov/Blast.cgi#alnHdr_WP_149492825)) | 19.80% |
| DddD (*Ruegeria pomeroyi* DSS-3) | orf01588 ([CoA transferase](https://blast.ncbi.nlm.nih.gov/Blast.cgi#alnHdr_WP_149492825)) | 23.62% |
| DddP (*Roseovarius nubinhibens* ISM) | orf00311 ([DUF3732 domain-containing protein)](https://blast.ncbi.nlm.nih.gov/Blast.cgi#alnHdr_WP_011512582) | 4.03% |
| DddP (*Roseobacter denitrificans* OCh 114) | orf02289 ([Methionine aminopeptidase](https://blast.ncbi.nlm.nih.gov/Blast.cgi#alnHdr_AGP48063)) | 6.83% |
| DddP (*Ruegeria pomeroyi* DSS-3) | orf01862 ([MATE family efflux transporter](https://blast.ncbi.nlm.nih.gov/Blast.cgi#alnHdr_WP_149408475)) | 4.90% |
| DddQ (*Ruegeria lacuscaerulensis* ITI-1157) | orf00350 ([1-deoxy-D-xylulose-5-phosphate synthase](https://blast.ncbi.nlm.nih.gov/Blast.cgi#alnHdr_WP_149492468)) | 8.64% |
| DddQ (*Ruegeria pomeroyi* DSS-3) | orf01055 ([Rod shape-determining protein RodA](https://blast.ncbi.nlm.nih.gov/Blast.cgi#alnHdr_WP_011513816)) | 7.53% |
| DddY (*Alcaligenes faecalis*) | orf01725 (N[itrite reductase small subunit NirD](https://blast.ncbi.nlm.nih.gov/Blast.cgi#alnHdr_WP_020443516)) | 3.78% |
| DddW (*Ruegeria pomeroyi* DSS-3) | orf02427 ([TRAP transporter small permease](https://blast.ncbi.nlm.nih.gov/Blast.cgi#alnHdr_WP_011514570)) | 9.14% |
| DddL (*Rhodobacter sphaeroides* 2.4.1) | orf02192 ([dTDP-glucose 4,6-dehydratase](https://blast.ncbi.nlm.nih.gov/Blast.cgi" \l "alnHdr_WP_149493016" \o "Go to alignment for dTDP-glucose 4,6-dehydratase [Psychrobacter sp. ANT_H56B] >gb\|KAA0924184.1\| dTDP-glucose 4,6-dehydratase [Psychrobacter sp. ANT_H56B])) | 6.94% |
| DddL (*Sulfitobacter* sp. EE-36) | orf01537 (Cupin domain-containing protein) | 7.96% |
| DddK (*Candidatus Pelagibacter ubique* HTCC1062) | orf01209 ([LysR family transcriptional regulator](https://blast.ncbi.nlm.nih.gov/Blast.cgi" \l "alnHdr_WP_020443718" \o "Go to alignment for MULTISPECIES: LysR family transcriptional regulator [Psychrobacter] >gb\|AGP49165.1\| LysR family transcriptional regulator [Psychrobacter sp. G] >gb\|ASE25543.1\| LysR family transcriptional regulator [Psychrobacter cryohalolentis])) | 16.47% |
| Alma1 (*Emiliania huxleyi*) | orf00316 ([Molybdenum cofactor biosysynthesis protein](https://blast.ncbi.nlm.nih.gov/Blast.cgi#alnHdr_AGP47838)) | 5.77% |

**Supplementary file 1c.** Kinetic parameters of DMSP lyases and DMSP demethylase DmdA.

| Protein | Organism | *K*_m_ for DMSP (mM) | *k*_cat_ for DMSP (s^-1^) | *k*_cat_/*K*_m_ (M^-1^ s^-1^ ) | Reference |
| --- | --- | --- | --- | --- | --- |
| DddX | *Psychrobacter* sp. D2 | 0.4 ± 0.03 | 0.7 ± 0.02 | 1.6 × 10^3^ | **This study** |
| DddY | *Desulfovibrio acrylicus* W218 | 0.4 | ND* | ND | ***van der Maarel et al., 1996*** |
|  | *Alcaligenes* sp.M3A | 1.4 | ND | ND | ***de Souza and Yoch, 1995*** |
|  | *Acinetobacter bereziniae* | 5.0 ± 0.6 | 8.3 ± 0.5 × 10^3^ | 1.7 × 10^6^ | ***Li et al., 2017*** |
| DddK | *Pelagibacter ubique* HTCC1062 | 3.7 ± 0.6 | 0.9 ± 0.1 | 2.4 × 10^2^ | ***Peng et al., 2019*** |
| DddW | *Ruegeria pomeroyi* DSS-3 | 8.7 ± 0.7 | 18.3 | 2.1 × 10^3^ | ***Brummett et al., 2015*** |
| Alma1 | *Emiliania huxleyi* | 9.0 ± 0.9 | 7 ± 0.3 × 10^2^ | 7.8 ± 0.6 × 10^4^ | ***Alcolombri et al., 2015*** |
| DddP | *Roseovarius nubinhibens* ISM | 13.8 ± 5.5 | 0.3 ± 0.1 | 18.7 | ***Kirkwood et al., 2010*** |
|  | *Ruegeria lacuscaerulensis* ITI_1157 | 17.1 ± 1.0 | ND | ND | ***Wang et al., 2015*** |
| DddQ | *Ruegeria lacuscaerulensis* ITI_1157 | 21.5 ± 6.8 | 1.0 ± 0.3 | 46.5 | ***Li et al., 2014*** |
| DddD | *Marinomonas* sp. MWYL1 | >40** | 12.6 ± 1.5 | 318 | ***Alcolombri et al., 2014*** |
| DddL | *Sulfitobacter* sp. EE-36 | ND | ND | ND | ***Curson et al., 2008*** |
| DmdA | *Ruegeria lacuscaerulensis* ITI_1157 | 4.1 ± 0.4 | ND | ND | ***Shao et al., 2019*** |
|  | *Ruegeria pomeroyi* DSS-3 | 5.4 ± 2.3 | 2.4 | 450 | ***Reisch et al., 2008*** |
|  | *Pelagibacter ubique* HTCC1062 | 13.2 ± 2.0 | 8.1 | 618 | ***Reisch et al., 2008*** |
|  | *Roseovarius nubinhibens* ISM | 35.7 ± 2.7 | ND | ND | ***Shao et al., 2019*** |

*No data available. **Saturation was not observed at 40 mM.

**Supplementary file 1d.** Crystallographic data collection and refinement parameters of DddX.

| Parameters | Se-derivative of DddX | DddX/ATP complex |
| --- | --- | --- |
| **Diffraction data** |  |  |
| Space group | *P*2_1_2_1_2_1_ | C2 |
| Unit cell |  |  |
| a, b, c (Å) | 85.3, 87.5, 327.6 | 143.6, 88.8, 273.3 |
| α, β, γ (°) | 90.0, 90.0, 90.0 | 90.0, 99.9, 90.0 |
| Resolution range (Å) | 50.0-3.3 (3.36-3.30) * | 50.0-2.25 (2.29-2.25) |
| Redundancy | 12.5 (11.6) | 3.2 (3.3) |
| Completeness (%) | 99.8 (98.4) | 97.3 (95.9) |
| *R*_merge_** | 0.1 (0.4) | 0.1 (0.4) |
| *I*/σ*I* | 18.0 (3.9) | 13.1 (2.0) |
| **Refinement statistics** |  |  |
| R-factor |  | 0.23 |
| Free R-factor |  | 0.18 |
| RMSD from ideal geometry |  |  |
| Bond lengths (Å) |  | 0.008 |
| Bond angles (°) |  | 1.1 |
| Ramachandran plot (%) |  |  |
| Favored |  | 93.7 |
| Allowed |  | 5.1 |
| Outliers |  | 1.2 |
| Overall B-factors (Å^2^) |  | 42.4 |

*Numbers in parentheses refer to data in the highest-resolution shell.

***R*_merge_=∑*_hkl_*∑*_i_*|*I*(*hkl*)*_i_* -<*I*(*hkl*)>|/∑*_hkl_*∑*_i_I*(*hkl*)*_i_*, where *I* is the observed intensity, <*I*(*hkl*)> represents the average intensity, and *I*(*hkl*)*_i_* represents the observed intensity of each unique reflection.

**Supplementary file 1e.** Homology alignment of proteins in *Psychrobacter* sp. D2 with known enzymes involved in acrylate catabolism.

| **Enzymes involved in acryloyl-CoA catabolism** | **Proteins of the highest similarity in *Psychrobacter*.** **sp.** **D2** | **Locus tag** | **Similarity** |
| --- | --- | --- | --- |
| AcuH (*Ruegeria pomeroyi* DSS-3) | orf01810 (Enoyl-CoA hydratase/isomerase family protein) | H0262_08710 | 45.58% |
|  | orf01692 (Enoyl-CoA hydratase/isomerase family protein) | H0262_08180 | 34.33% |
|  | orf01695 (Enoyl-CoA hydratase/isomerase family protein) | H0262_08190 | 32.48% |
| AcuI (*Ruegeria pomeroyi* DSS-3) | orf00105 (acryloyl-CoA reductase) | H0262_00540 | 40.57% |
|  | orf02674 (acryloyl-CoA reductase) | H0262_12930 | 36.15% |
| AcuN (*Halomona*s sp. HTNK1) | orf01588 ([CoA transferase](https://blast.ncbi.nlm.nih.gov/Blast.cgi#alnHdr_WP_149492825)) | H0262_07715 | 31.58% |
| AcuK (*Halomona*s sp. HTNK1) | orf01810 (Enoyl-CoA hydratase/isomerase family protein) | H0262_08710 | 44.00% |
| PrpE (*Ruegeria pomeroyi* DSS-3) | orf01091 (propionate--CoA ligase) | H0262_05290 | 41.98% |

**Supplementary file 1f.** Strains and plasmids used in this study.

| **Strains** | **Description** | **Source** |
| --- | --- | --- |
| *Psychrobacter* sp. D2 | Wild-type isolate | This study |
| Δ*dddX* | the *dddX* gene deletion mutant | This study |
| Δ*dddX*/pBBR1MCS-*dddX* | Δ*dddX* containing pBBR1MCS-*dddX* plasmid | This study |
| Δ*dddX*/pBBR1MCS | Δ*dddX* containing pBBR1MCS plasmid | This study |
| *Escherichia. coli* WM3064 | RP4 (tra) in chromosome, DAP^-^, 37°C | (***Dehio et al., 1997***) |
| *Escherichia. coli* DH5α | Used for gene cloning | Vazyme Biotech company (China) |
| *Escherichia. coli* BL21(DE3) | Used for gene expression | Vazyme Biotech company (China) |
| **Plasmids** |  |  |
| pK18*mobsacB*-Ery | pk18*mobsacB* containing the erythromycin resistant gene from pHT304, Kan^r^*, Ery^r^** | (***Wang et al., 2015***) |
| pK18Ery-*dddX* | pK18*mobsacB*-Ery containing the homologous arms of the *dddX* gene of *Psychrobacter*. sp. D2, Kan^r^, Ery^r^ | This study |
| pBBR1MCS | Broad-host-range cloning vector, Kan^r^ | (***Kovach et al., 1995***) |
| pBBR1MCS-*dddX* | pBBR1MCS containing the *dddX* gene and its promoter of *Psychrobacter*. sp. D2, Kan^r^ | This study |
| pET-22b | Used for gene expression | Novagen (Germany) |

* Kan^r^, kanamycin resistance.

** Ery^r^, erythromycin resistance.

**Supplementary file 1g.** Composition of the basal medium (lacking the carbon source).

| Solution* | Components |
| --- | --- |
| 1 | 0.05% (w/v) NH_4_Cl, 3% (w/v) NaCl, 0.3% (w/v) MgCl_2_·6H_2_O, 0.2% (w/v) K_2_SO_4_, 0.02% (w/v) K_2_HPO_4_, 0.001% (w/v) CaCl_2_, 0.0006% (w/v) FeCl_3_·6H_2_O, 0.0005% (w/v) Na_2_MoO_4_·7H_2_O, 0.0004% (w/v) CuCl_2_·2H_2_O, 0.6% (w/v) Tris. [1.5% (w/v) agar for solid medium] |
| 2 (***Kanagawa et al., 1982***) | 0.001% (w/v) thiamine·HCl, 0.002% (w/v) nicotinic acid, 0.002% (w/v) pyridoxine·HCl, 0.002% (w/v) riboflavin, 0.0001% (w/v) biotin, 0.0001% (w/v) cyanocobalamin, 0.001% (w/v) *p*-aminobenzoic acid, 0.002% (w/v) calcium pantothenate. |

* Solution 1 was autoclaved at 121°C for 20 min. Solution 2 was filter-sterilized before it was combined with solution 1.

**Supplementary file 1h.** Primers used in this study.

| **Primers** | **Sequence (5’-3’)** | **Purpose** |
| --- | --- | --- |
| 27F | AGAGTTTGATCCTGGCTCAG | Amplification of cultivated strains 16S rRNA genes (***Lane et al., 1985***) |
| 1492R | GGTTACCTTGTTACGACTT |  |
| RT-*1696*-F | GTTGTCTGTTACTGGATT | Used for RT-qPCR of the *1696* gene |
| RT-*1696*-R | AGGTAGTCGCTAAGAATA |  |
| RT-*dddX*-F | GACCGCTATGGAGAAGTA | Used for RT-qPCR of the *dddX* gene |
| RT-*dddX*-R | CAAGACATCAAGTGCTACC |  |
| RT-*1698*-F | ATGGTTGGCGTCAATATC | Used for RT-qPCR of the *1698* gene |
| RT-*1698*-R | CAAGTCGGCATAGAGAAC |  |
| RT-*1699*-F | CGAATACCTAGCCTAGAAGAGA | Used for RT-qPCR of the *1699* gene |
| RT-*1699*-R | TAATGAGCGATCCATACTATTGTC |  |
| RT-*recA*-F | CTTCTGTGCGTATGGATATTC | Used for RT-qPCR of the *recA* gene |
| RT-*recA*-R | TGCCTTCACCGTAAGTAAT |  |
| *dddX*-UP-F | GTAAAACGACGGCCAGTGCCAAGCTTTAATAGCTTCAGTCCACGTTTC | Upstream homologous fragment of the *dddX* gene |
| *dddX*-UP-R | CTTATTCAATAGAAATTATACAAGCGTTTATACATTATAT |  |
| *dddX*-Down-F | ATATAATGTATAAACGCTTGTATAATTTCTATTGAATAAG | Downstream homologous fragment of the *dddX* gene |
| *dddX*-Down-R | GTCATAAGATTAGTCACTGGGGATCCAGGCAAACGCTGTCAGGCGCTC |  |
| *dddX*-1000-F | AACTAAAATTTGAAAACTCAGGCTTTTC | Confirmation of the Δ*dddX* mutant |
| *dddX*-1000-R | TGAATATGGTAATGGTACTTGTATTTATAC |  |
| *dddX*-300Up-F | CAGATGGCAACAATCAAAACAAATGTAGAGATGAAGATGAAC |  |
| *dddX*-700Down-R | TGCCGATCAAGGTGCTAAACTGGTCATCGATGGCTCTGATG |  |
| *dddX*-pBBR1-PF | CGGGGTACCTTATAAATTATAGATGACAATGATAG | Complementation of the Δ*dddX* mutant |
| *dddX*-pBBR1-PR | CCGCTCGAGTTTAATTACTCCTTCCTTAGAGTTAAC |  |
| *dddX*W-F | AAGAAGGAGATATACATATGTTGACTGGTCAGATAATTGAG | Amplification of the *dddX* gene |
| *dddX*W-R | TGGTGGTGGTGGTGCTCGAGCACCAGCTCACTACATTTTTTG |  |
